# The potential insights of Traditional Chinese Medicine on treatment of COVID-19

**DOI:** 10.1186/s13020-020-00326-w

**Published:** 2020-05-24

**Authors:** Tong Tong, Ying-Qi Wu, Wei-Jian Ni, Ai-Zong Shen, Sheng Liu

**Affiliations:** Department of Pharmacy, Anhui Provincial Hospital, The First Affiliated Hospital of USTC, Division of Life Sciences and Medicine, University of Science and Technology of China, Hefei, 230001 Anhui China

**Keywords:** Traditional Chinese medicine, SARS-CoV-2, COVID-19, Treatment, Mechanism

## Abstract

Corona Virus Disease 2019 (COVID-19) broke out in 2019 and spread rapidly around the world. There is still no specific antiviral therapy to the current pandemic. In China, historical records show that Traditional Chinese Medicine (TCM) is effective in prevention and enhancing the resistance to pandemic with unique insights. To fight with COVID-19, National Health and Commission of PRC has recommended some TCM in the guideline, such as HuoxiangZhengqi, LianhuaQingwen ShufengJiedu and XueBijing, and actually displayed a remarkable effect in clinical treatment strategic for COVID-19. We review studies to provide an in-depth understanding into the effect of TCM, and also introduce the possible mechanism involved in COVID-19 treatment.

## Background

Currently, the disease caused by the Severe Acute Respiratory Syndrome Coronavirus 2 (SARS-CoV-2) was named Corona Virus Disease 2019 (COVID-19), have been spread around the world with over 2,430,000 confirmed cases and nearly 160,000 deaths (up to 21 April). Notably, the situation is getting worse and worse.

By comparing the infection patterns of coronavirus hosts in other vertebrates, SARS-CoV-2 was found to be similar to those of SARS-CoV and Middle East Respiratory Syndrome Coronavirus (MERS-CoV), SARS-CoV-2 could also be transmitted among humans. It can infect human cells by utilizing human angiotensin converting enzyme 2 (ACE2) as a receptor. Clinical presentation of COVID-19 is fever, fatigue, and dry cough and some patients show with nasal congestion, runny nose, inappetence, diarrhea and pneumonia on computed tomography (CT). Some severe cases can rapidly develop into acute respiratory distress syndrome (ARDS), refractory metabolic acidosis, septic shock, and coagulation dysfunction [1].

Unfortunately, there is no current any specific antiviral treatment for patients with suspected or confirmed COVID-19. According to the experiences in the SARS treatment, National Health Commission (NHC) of the PRC recommended TCM as strategies for COVID-19 treatment. From the current results, TCM has exhibited positive effects in combating with COVID-19. This article reviews focuses on the effects of 4 TCM in COVID-19 treatment: HuoxiangZhengqi, LianhuaQingwen, ShufengJiedu and XueBijing, and summarize the mechanism of these drugs on COVID-19, to provide a deeper insight of therapeutic benefits of these TCM.

### HuoxiangZhengqi ameliorate symptoms through anti-inflammatory effects

HuoxiangZhengqi (HXZQ) formula contains almost ten Chinese herbs. It is applied to traditional syndrome differentiation for cold, fever, nausea and vomiting, abdominal distension, diarrhea, also showed good effects in pediatric in dyspepsia to achieve the harmony inside and outside, the efficacy of antiemetic diarrhea. In NHC guidelines, HXZQ was recommended for clinical features with aversion to cold with no sweating, headache, full chest diaphragm, urine frequency, yellow urine, etc.

According to the report from COVID-19 patients, the rise of creatine kinase (CK) and lactate dehydrogenase (LDH) was said to be related to lung cell damage and systemic symptoms [1]. In SARS treatment, HXZQ showed good effects in improving CK, LDH and oxygenation indexes [2]. It implied that HXZQ could improve oxygenation indexes and systemic symptoms through down-regulating the level of CK and LDH, might be a possible mechanism in treating COVID-19.

COVID-19 could cause strongly immune reaction. COVID-19 patients showed that in the peripheral blood inflammatory cytokines such as, IL-2, IL-6, IL-10 and Tumor Necrosis Factor α (TNF-α) increased and CD4^+^, CD8^+^, CD16^+^, CD19^+^ and CD45^+^ T cells were decreased [3], but an increase in Th17 cell proportion [4]. In animal model, HXZQ could regulate CD4^+^ and CD8^+^ cells and suppression on TNF-α level [5]. It indicates that HXZQ have the function of anti-inflammation and immune regulation in COVID-19 through suppress inflammatory factors and regulate immune response. In studies based on network pharmacology and molecular docking, researchers found that the core compounds of HXZQ such as quercetin, isorhamnetin, irisolidone, have a stronger binding ability to SARS-CoV-2 3CL (Mpro) than that of remdesivir with COVID-19. They could combine with ACE2 binding to PI3K-Akt signaling pathway to affect viral replication, thus exerting therapeutic effect on COVID-19. Which is worthy of further research and helps to provide theoretical guidance [6].

### LianhuaQingwen protect lung from pneumonia via inhibiting pro-inflammatory cytokines production

LianhuaQingwen (LHQW) formula is composed of 13 Chinese herbs, which was approved in the SARS treatment in 2003. It has become an effective treatment for SARS-CoV, MERS-CoV, H_1_N_1_, H_3_N_2_, and H_7_N_9_. Analysis of COVID-19 treatment with LHQW indicate that LHQW could significantly relieve clinical symptoms in patients with fever, weakness, cough and reduce the course of the COVID-19 [7]. The molecular docking results showed that the key components are kaempferol, quercetin, luteolin, glycyrrhetinic acid, stigmasterol, indigo had good binding ability with SARS-CoV-2 3CL (Mpro) and ACE2, acts on COVID-19 through multiple components, multiple targets, and multiple pathways [8].

Lung, which is the target organ of COVID-19, according to the TCM theory, “damp and toxin plague” is the main cause of COVID-19 etiology, even cause fatal pneumonia. Increasing evidence points out that cytokine storm displays a key role in causing fatal pneumonia [9]. In pulmonary oxidative lesions models, LHQW could significantly reduce pathological changes, including alveolar septum thickening, capillary congestion, interstitial edema, peripheral bronchial lymphocyte infiltration and neutrophils, the mechanism might be related to the levels of malonaldehyde, LDH, glutathione peroxidase, and super oxide dismutase were regulated by LHQW, which play significant role in pathogenesis of lung injury [10]. LHQW could inhibits the replication of SARS-COV-2 in vitro, and significantly reduced pro-inflammatory cytokines production (IL-6, TNF-α), which might mediate strong immune response or even cytokine storm [11].

In children with mycoplasma pneumoniae pneumonia, after treatment with LHQW, the CD3^+^, CD4^+^, and CD8^+^ T cell subsets in patients were significantly altered, and IL-6, c-reactive protein (CRP) in serum and procalcitonin (PCT) levels significantly reduced [12]. In COVID-19 patients treat with LHQW, the total effective rate was 74.55%, and 28 patients were cured after 3 days. After 7 days of treatment, the total effective rate was 92.73% and 39 patients were totally cured, main symptoms patients experienced including fever, cough, fatigue and chest tightness were significantly reduced [7].

### ShufengJiedu act on COVID-19 through multiple targets and multiple inflammatory signaling pathways

The main components of ShufengJiedu (SFJD) are polygonum cuspidatum, forsythia, radix isatidis, bupleurum root, rhizoma corydalis, verbena, reed root, liquorice. Previous research suggested that SFJD can alleviate the clinical symptoms of patients with Acute Exacerbation of Chronic Obstructive Pulmonary Disease (AECOPD) and shorten the hospitalization time [13]. SFJD not only had the function of inhibiting virus proliferation and anti-inflammatory, but also has certain immune regulation function. Whatever in vivo or vitro, SFJD had a function of inhibiting airway inflammatory responses via regulating NLRP3 inflammasome and then down-regulating the level of IL-18 and IL-1β which similar to these effects of oseltamivir [14].

SFJD combined with western medicine treatment in COVID-19 have been gained significant improvement in pneumonia associated symptoms [15]. The combination of SFJD and Arbidol was better than Arbidol alone in the treatment of COVID-19, which could significantly shorten the symptoms improvement time and negative conversion time of the clinical patients [16]. In deeper studies, after SFJD treatment, partial pressure of oxygen in lung tissue increased, the level of lactic acid decreased, inflammatory cytokines such as IL-1β, IL-6 and TNF-α were inhibited [17]. Latest molecular docking results showed that quercetin, kaempferol, luteolin, these core compounds in SFJD had high affinity with target proteins. Similar to LHQW, these chemical compounds involved a variety of biological processes and pathways to treat COVID-19 by combining with key target proteins IL-6, ALB, and MAPK3, which supported the clinical application with COVID-19 [18].

### XueBijing injection reduce multiple organ damage caused by COVID-19 through anti-inflammation and improving immune function

With the approving for marketing in 2004 by Chinese authorities, Xuebijing (XBJ) injection has been used in H1N1, H7N9, dengue fever, MERS as well as ebola. From previous report, XBJ could antagonize endotoxin, anti-inflammation, improving immune function and microcirculation, and regulating coagulation disorders [19, 20]. COVID-19 patients often occur respiratory distress, coagulation disorders and microcirculation disorders, especially in patients with systemic inflammatory response syndrome or/and multiple organ failure, timely use of XBJ can effectively reverse the situation and reduce the fatality. Currently, Chinese researchers are now conducting a prospective analysis of the clinical efficacy of XBJ on COVID-19. Hydroxysafflor yellow A, chlorogenic acid and salvianolic acid B were major compositions in XBJ by molecular docking [21], through “multi-component, multi-target, multi-pathway” to play the role of anti-inflammatory, vascular endothelial protection and immunity. XBJ could inhibit inflammatory cytokines such as IL-1, IL-6, IL-8, IL-17 and TNF-α [22]. By increasing the Th1/Th2 ratio, XBJ injection could improve the proportion of Th1 cells in septic rats [23], promote the apoptosis of CD4^+^ CD25^+^ T cells (Tregs) [24, 25], and further improve the immune function.

### Potential mechanism of 4 TCM in COVID-19 treatment

To date, NHC has issued 7 editions guidelines of diagnosis and treatment for COVID-19. In each edition, TCM has been recommended for COVID-19 treatment based on different stage and symptom differentiation. TCM has shown good effects in combating with COVID-19, early intervention of TCM in COVID-19 treatment could increase cure rate, shorten disease course and reduce mortality cases. According to the guidelines, 4 TCM in this paper and main ingredients and traditional indications versus COVID-19 are as follow (Tables 1, 2).

**Table 1:** 4 TCM recommended by guidelines of treatment for COVID-19

	Stage of disease	Symptom
HXZQ	Medical observation period	Hypodynamia with gastrointestinal upset
LHQW	Medical observation period	Hypodynamia with fever
SFJD	Medical observation period	Hypodynamia with fever
XBJ	Clinical treatment period	Several cases and critical cases

**Table 2:** Main ingredients and traditional indications versus COVID-19

	Main ingredients	Traditional indications	COVID-19
HXZQ	Ageratum, poriacocos, perilla, angelica, orange peel, radix platycodonis, atractylodes, magnolia officinalis, pinellia, liquorice	Gastrointestinal cold, influenza and upper respiratory tract infections, viral enteritis, diarrhea	Hypodynamia accompanied by gastrointestinal upset, cold without sweating, headache and heaviness, limb pain, thirst with no desire to drink, yellow urine, frequent micturition
LHQW	Forsythia, honeysuckle, ephedra, male fern rhizome, houttuyniae, pogostemon cablin, rheum, rhodiola, menthol, liquorice	Fever, aversion to cold, muscular soreness, stuffy nose runny nose, cough, headache, pharyngoxerosis and pharyngalgia	Hypodynamia and fever
SFJD	Polygonum cuspidatum, forsythia, isatidis, bupleurum, rhizoma corydalis, verbena, reed root, liquorice	Fever, aversion to wind, pharyngalgia, headache, stuffy nose runny nose, cough	Hypodynamia and fever
XBJ	Paeoniae, angelica rhizoma Chuanxiong, flos carthami, salviae miltiorrhizae	Fever, dyspnea, palpitation, dysphoria, infection, viscera damage	Dyspnea, high fever or alternating cold and heat, cough with less phlegm, coma, etc.

**Fig. 1:**
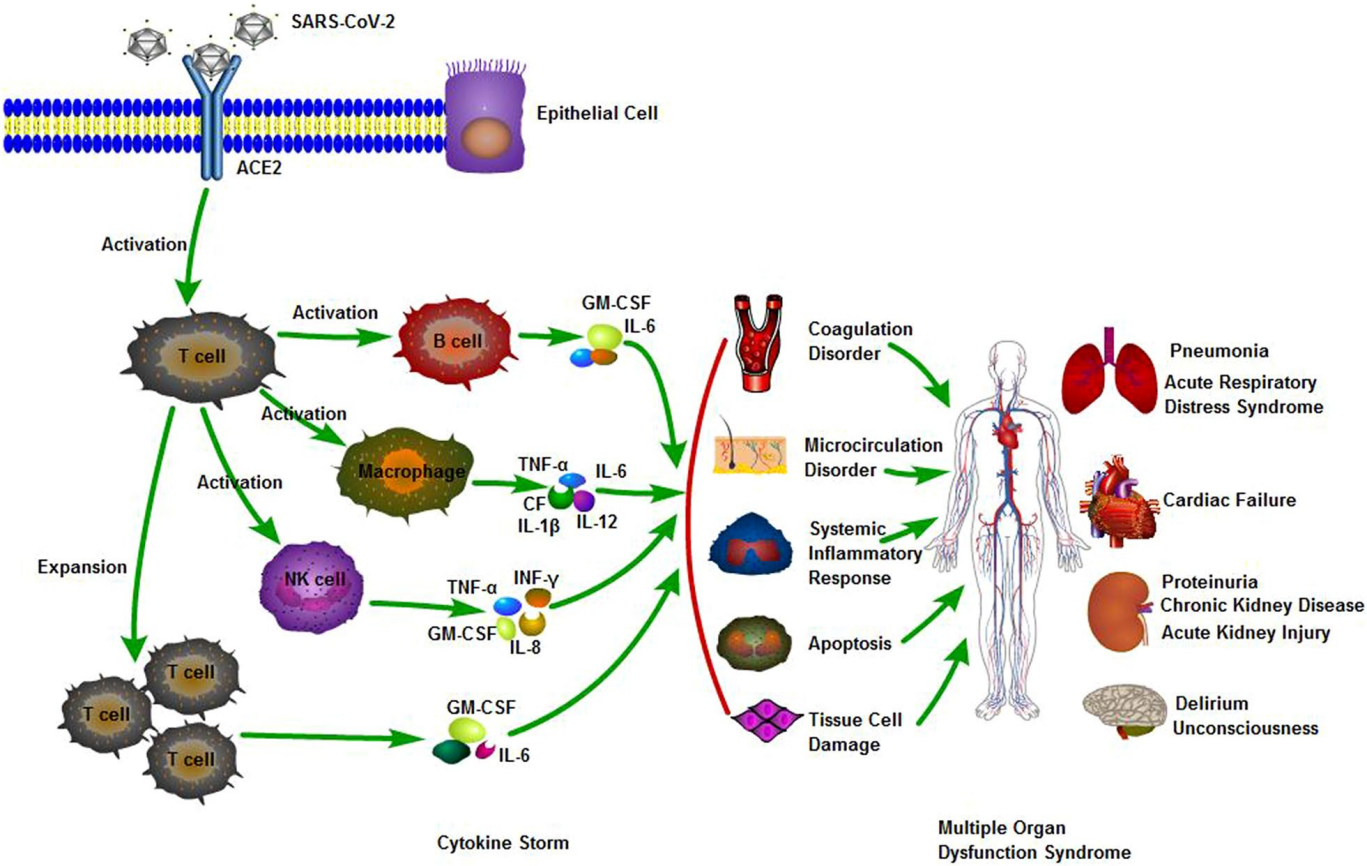
Pathogenesis of COVID-19. SARS-CoV-2 is binding to ACE2 receptor via infecting epithelial cell, with the activation of immune cell, they release a large number of cytokines, and then produce cytokine storm, resulting in MODS

So far as we know, COVID-19 could cause mortal systemic complication with strongly immune response or cytokine storm, further cause multiple organ dysfunction syndrome (MODS), which is the main cause of mortality in COVID-19 (Fig. 1).

**Fig. 2:**
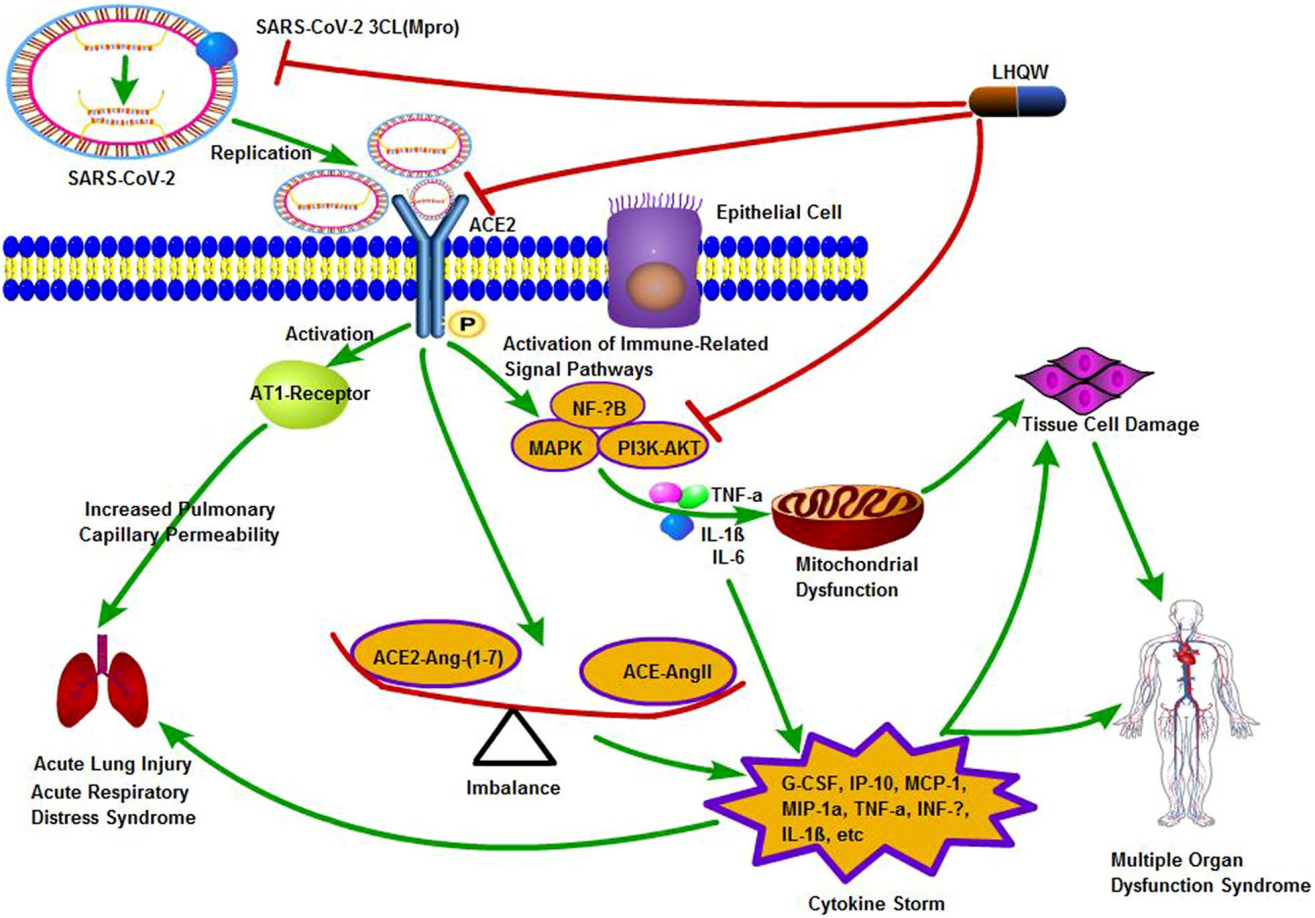
Mechanism of LHQW in treating with COVID-19. LHQW exhibit functions on COVID-19 via “multi-component, multi-target, multi-pathway”. Firstly, major chemical constituents in LHQW could combine with SARS-CoV-2 3CL (Mpro), inhibiting the SARS-COV-2 replication. Secondly, there is an imbalance of ACE-Ang-II and ACE2-Ang-(1-7), which can lead to overwhelming pro-inflammatory cytokines with cytokine storm, LHQW could regulate balance. Thirdly, LHQW could regulate immune-related signal pathway (MAPK, NF-κB, PI3K-AKT, ect), reduce the production of pro-inflammatory cytokines

TCM can regulate the inflammatory response of the body through “multi-component, multi-target, multi-pathway” to improve the immunity of the body, so as to ameliorate symptoms, prevent complications, and achieve indirect suppression of the virus. Through the prediction of molecular docking, major chemical constituents and possible targets of 4 TCM in COVID-19 were found (Table 3). According to the analysis and illumination from the latest literatures, we summarized possible mechanism and related targets of LHQW in treating with COVID-19 and showed it in Fig. 2.

**Table 3:** Major chemical constituents and possible targets of TCM in COVID-19

	Major chemical constituents	Possible targets	References
HXZQ	Quercetin, isorhamnetin, irisolidone, kaempferol, wogonin, baicalein	PTGS2, HSP90AB1, CAMSAP2	[5, 6]
LHQW	Kaempferol, quercetin, glycyrrhetinic acid, stigmasterol, indigo	SARS-CoV-2 3CL (Mpro), ACE2, MAPK, PI3K-AKT, NF-κB	[8]
SFJD	Quercetin, kaempferol, luteolin	IL-6, ALB, MAPK3	[18]
XBJ	Hydroxysafflor yellow A, chlorogenic acid, salvianolic acid B	NF-κB, HIF-1, VEGF	[21]

## Conclusion

With the emergence of COVID-19, three cases of zoonotic coronavirus disease have been identified in this century. However, COVID-19 has caused more deaths to date than SARS and MERS. Accumulated experiences from thousands of years in the treatment of epidemic, TCM is worth learning. TCM has holistic therapy theory, it could balance Yin and Yang, enhancing human body resistance to eliminate epidemic factors. That’s why China government recommended TCM in combating COVID-19 timely.

Although, laboratory studies on the effect of TCM are far behind the clinical application in COVID-19 treatment, further studies in molecular mechanisms are expected to clarify the effect of TCM on COVID-19. In this study, combining with the latest research, this work highlights the prospect of therapeutic effects and mechanism of 4 TCM in COVID-19 treatment. The therapeutic effects of 4 TCM in COVID-19 potentially focus on: anti-inflammatory, inhibiting pro-inflammatory cytokine production and cut off cytokine storm, regulating immune response, protecting organ damage. By the continuing expansion of this pandemic, we anticipate more and more good messages about anti-SARS-CoV-2 activity of TCM will be discovered to benefit with COVID-19 patients and finally overcome the current epidemic around the corner.

## Data Availability

Not applicable.

## Abbreviations

COVID-19: Corona virus disease 2019
TCM: Traditional Chinese Medicine
CT: Computed tomography
ARDS: Acute respiratory distress syndrome
SARS-CoV-2: Severe acute respiratory syndrome coronavirus 2
ACE2: Angiotensin-converting enzyme 2
MERS-CoV: Middle east respiratory syndrome coronavirus
NHC: National health commission
HXZQ: HuoxiangZhengqi
CK: Creatine kinase
LDH: Lactate dehydrogenase
TNF-α: Tumor necrosis factor α
LHQW: LianhuaQingwen
IL: Interleukin
CRP: c-reactive protein
PCT: Procalcitonin
SFJD: ShufengJiedu
AECOPD: Acute exacerbation of chronic obstructive pulmonary disease
ERK: Extracellular regulated protein kinases
TGF-β: Transforming growth factor-β
NF-κB: Nuclear factor-κb
MAPK: Mitogen activated protein kinase
XBJ: Xuebijing
PI3K-AKT: Phosphatidylinositol 3-kinase protein kinase B
PTGS2: Prostaglandin-endoperoxide synthase 2
HSP90AB1: Heat shock protein 90 kDa alpha B1
CAMSAP2: Calmodulin-regulated spectrin-associated proteins 2
HIF: Hypoxia inducible factor
VEGF: Vascular endothelial growth factor
